# Multiview three-dimensional reconstruction by millimetre-wave portable camera

**DOI:** 10.1038/s41598-017-06475-7

**Published:** 2017-07-25

**Authors:** Jaime Laviada, Ana Arboleya-Arboleya, Yuri Álvarez, Borja González-Valdés, Fernando Las-Heras

**Affiliations:** 1 Universidad de Oviedo, Dept. Ingeniería Eléctrica, Gijón, 33203 Spain; 2 Universidad de Vigo, Dept. Teoría de la Señal y Comunicaciones, Vigo, 36310 Spain

## Abstract

Millimetre-wave imaging is a powerful non-destructive inspection technique which has become widely used in areas such as through-the-wall imaging or concealed weapon detection. Nevertheless, current systems are usually limited to either a single view point providing a limited 3D millimeter-wave model or a multiview relying on the accurate movement of a robot arm through precise positions resulting in very bulky systems. In this paper, we present a set of techniques to achieve a multiview millimetre-wave scanner. The aperture of the scanner is kept below 16 cm so it can be portable and, consequently, multiview can be achieved by simple hand movements. In addition, optical images are also acquired with a two-fold purpose: i) building a complementary 3D-model by employing Structure from Movement (SfM) techniques; ii) estimating the scanner position and poses. The proposed technology is illustrated for people screening, proving the capacity of the system to detect hidden weapons.

## Introduction

Properties of the electromagnetic (EM) waves along the EM spectrum (i.e., X-rays, visible light, infrared, terahertz, microwaves, etc.) are extremely heterogeneous enabling a wide variety of applications. Among these portions of the spectrum, millimetre-waves (mm-waves), covering the range from 30 GHz to 300 GHz, provide a good trade-off between penetration capabilities and wavelength size. Consequently, they are widely used for EM imaging as they can be employed to generate images with resolution similar to conventional optical images. For this reason, mm-waves have become very appealing for fields such as security^1, 2^ or non-destructive evaluation^3, 4^. Furthermore, they lay in the non-ionizing area of the EM spectrum and, consequently, they are harmless for human beings.

There is a large variety of mm-wave imagers based on different strategies. For example, it is usual to classify the systems as passive or active depending on if they require emitting or not some kind of wave. Among *passive systems*, radiometers are able to capture the spontaneous radiation of bodies at a given temperature by using high gain antennas^5, 6^ so that information of small spots can be collected yielding the final image. In general, these setups have been used to image in the range of tens of metres due to the distance required by the involved large antennas to focus the scene. Focal plane arrays (FPAs), comprising a set of lenses and an array of receivers, are also able to capture spontaneous radiation but their working principle is similar to a conventional photography camera^7, 8^. Nevertheless, the use of these imagers requires large frequency bandwidths in order to collect enough radiation for retrieving the image. Thus, they are mostly deployed at submm-wave bands where larger bandwidths are available.

*Active systems*, based on illuminating the object under test by a certain source and capturing the reflected power, are also widespread^9^. As in the case of radiometers, active systems based on high gain antennas have been demonstrated^10^. Active imaging can also be done at ranges of a few centimetres by resorting to near focusing lenses^3^ that are mechanically moved to perform a raster scan. Finally, real-time imaging at distances in the order of a meter has also been validated by synthetic aperture radar (SAR) techniques combined with hybrid electronic/mechanic^1^ or fully electronic^11^ approaches.

Mm-wave technology has been boosted in the last years due to these imaging applications as well as others such as high capacity radiolinks or automotive radar. Consequently, a wide variety of Monolithic Microwave Integrated Circuits (MMIC) is available reducing the cost and easing the implementation of mm-wave setups. These advances in mm-wave technology have enabled the implementation of the first generation of portable mm-wave scanners^12^ and fully electronic cameras^13–15^.

Independently of the imaging strategy, EM scanners usually consider data acquisition from a single point of view. Although the images can provide an astonishing degree of quality, even containing information relative to depth, this kind of acquisition is limited by its own nature. For example, when scanning a human being, information from the backward view is completely occluded forcing the use of a second scanning panel behind the subject under test. In short-range imaging, active mm-wave scanners, which usually comprise transmitters and receivers placed very close to each other (i.e., a monostatic or quasimonostatic setup), suffer from problems when the target is not illuminated by a wave normal to its surfaces. Since the impinging waves are bounced into a direction which is different from the transmitting/receiving direction, only a small fraction of the reflected energy is received^16, 17^. This problem is alleviated by multistatic scanners^11, 18^, with dissociated transmitters and receivers positions, enabling a wider observation range. Other alternatives, including commercial systems, employ cylindrical scanning based on the accurate movement of a robot arm^19^.

Last generation of scanners tries to bypass the aforementioned difficulties. For example, a walk-through imaging system has been presented so that it can consider snapshots at different positions in order that multiple views are available. However, each view is independently considered and not merged into a global model^20, 21^. Each view can be independently considered^20, 21^ or merged into a global model^22^. Other authors have focused on reducing the complexity of the required electronic by resorting to metasurface antennas with capacity to produce a wide set of radiation patterns by a simple frequency sweep^23^.

On other side, conventional optical cameras have been able to benefit from multiview acquisitions for many years, enabling the possibility of building three-dimensional (3D) models or photogrammetry^24^. This kind of techniques can be implemented either by a multicamera-setup, where the positions are accurately known, or by Structure from Motion (SfM)^25^ techniques where the camera (or, alternatively the object) is arbitrarily moved and, consequently, the position is also estimated from the images themself. Furthermore, it has been demonstrated in the recent years that the algorithms supporting this technique can run on real-time even on (relatively) low performance devices such as smartphones^26^.

The goal of this paper is to demonstrate the possibility of multiview with a mm-wave camera for short-range imaging. Thus, 3D images benefiting from the penetration capabilities of the EM waves can be calculated. Furthermore, working frequency and bandwidth match those of available commercial solutions which exploit relatively inexpensive MMIC for automotive radar to build the RF system. It is also relevant to mention that the positioning of the camera is based on the information extracted from a conventional camera by resorting to SfM. Thus, a 3D conventional model is also simultaneously built resulting in a more insightful inspection system.

## Multiview mm-wave camera

**Figure 1:**
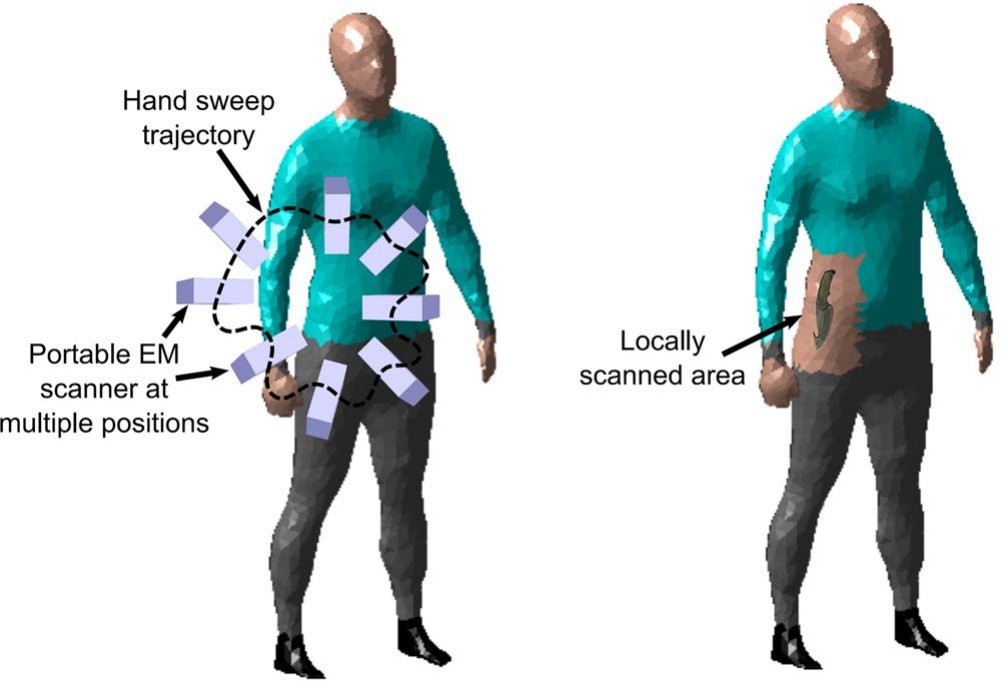
Application example for locally scanning in search of concealed weapons.

Let us consider Fig. 1 to illustrate the working principle of the proposed approach. In this example, subject under test is locally scanned, in contrast to full body scanners, to inspect a specific suspicious area. For this purpose, the portable mm-wave scanner with an attached optical camera is arbitrarily moved along different positions around the area to be scanned.

During the sweep, EM data are acquired from different positions. According to the state of the art devices, these data can be collected by fully electronic devices^11, 14, 15^. Nevertheless, in the proof of concept presented in this paper, this acquisition is performed by means of raster scanning with a single sensor. In parallel to the EM acquisition, optical images are also captured from, at least, the same positions so that the scanner attitudes and positions as well as a 3D model can be estimated by resorting to SfM. These data are assembled on-the-fly assembled to build two complementary 3D models based on the EM and optical data.

**Figure 2:**
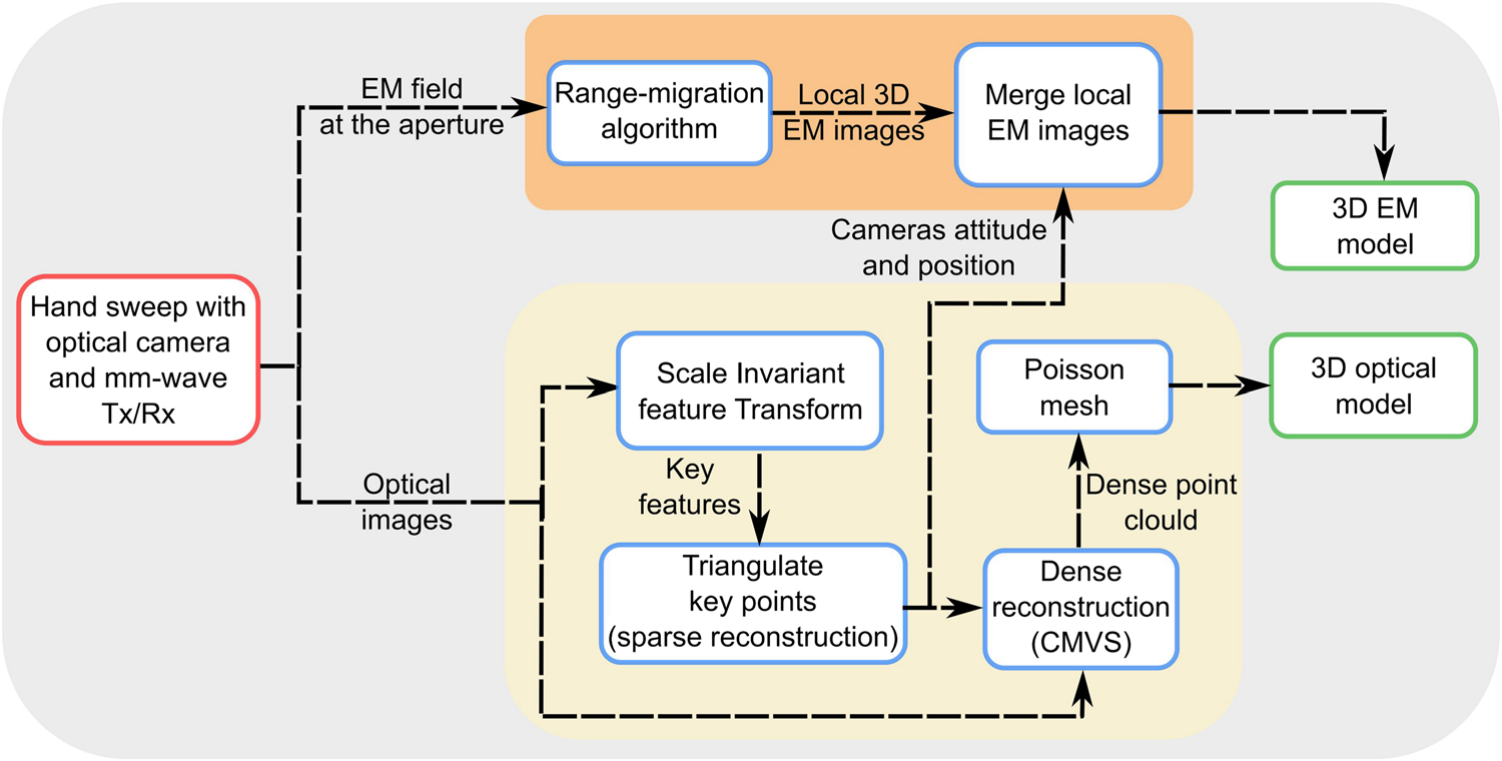
Global diagram combining computer vision techniques with EM imaging.

The different steps required for the processing of the multiview mm-wave camera are illustrated in Fig. 2 and detailed in the following sections.

## MM-wave active imaging

As pointed out in the introduction, the most widespread strategy for real-time NF short-range imaging is the use of active scanners based on SAR techniques. Unlike passive scanners, which produce images according to the natural emissivity of the elements in a given scene, the images of these active scanners contain information about the *reflectivity* of the scene. In order to apply SAR techniques, a set of transmitters and receivers are sequentially activated. The conventional topology comprises a monostatic setup with a set of independent transceivers equally spaced along a *two-dimensional aperture* ^1^.

**Figure 3:**
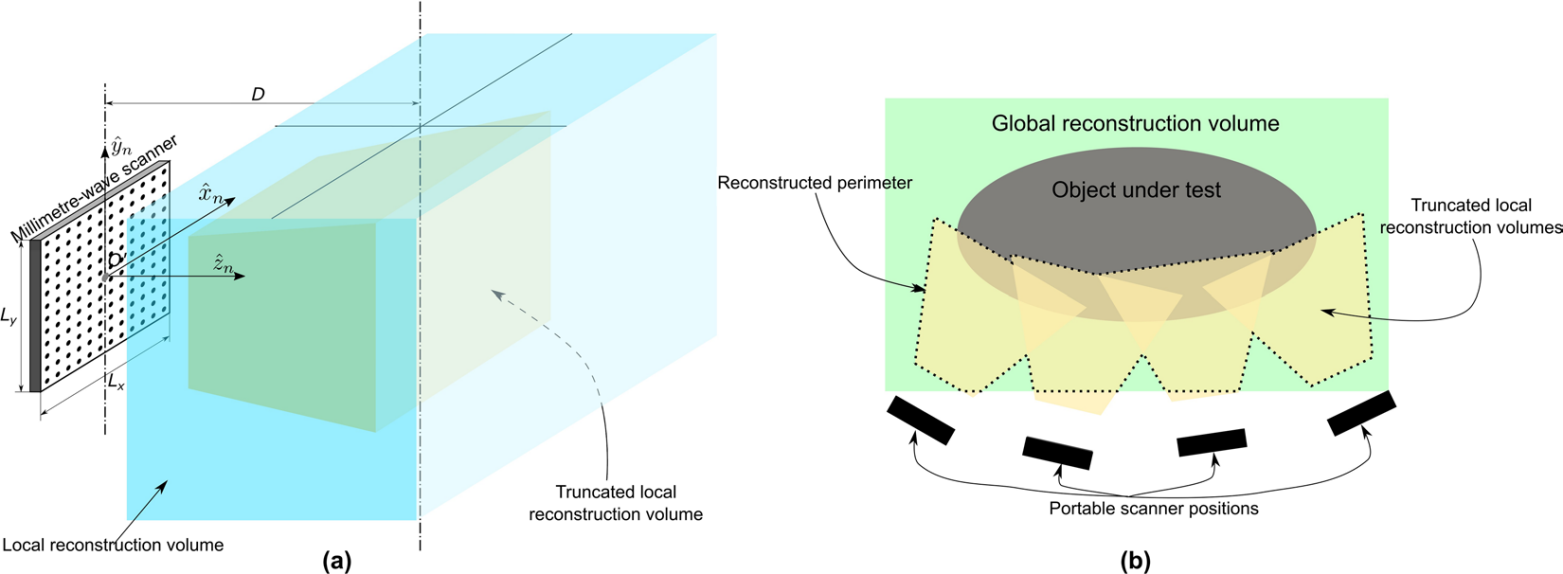
Reconstruction volumes involved in the proposed approach: (**a**) local reconstruction volumes and their corresponding truncated volume; (**b**) Global reconstruction volume filled by merging the reflectivities computed in the truncated local reconstruction volumes.

This kind of arrangement can be easily processed in real-time by applying *range-migration* techniques based on efficient Fourier transforms. Let us consider the scanner at the *n*-th position, so that a local system of coordinates can be defined by the vectors ${\hat{x}}^{(n)}$, ${\hat{y}}^{(n)}$ and ${\hat{z}}^{(n)}$ (see Fig. 3a). As detailed in refs 1, 27, the reflectivity *ρ* at a given point, expressed in the local coordinates (*x* ^(*n*)^, *y* ^(*n*)^, *z* ^(*n*)^), can be efficiently computed as:

1 $$\rho ({x}^{(n)},{y}^{(n)},{z}^{(n)})={ {\mathcal F} }_{3D}^{-1}\{{ {\mathcal F} }_{2D}\{ {\mathcal F} ({x}^{(n)},{y}^{(n)},\omega )W({x}^{(n)},{y}^{(n)},\omega )\}{e}^{-jD\sqrt{\mathrm{4(}\omega /c{)}^{2}-{k}_{x}^{2}-{k}_{y}^{2}}}\},$$

where *f*(*x* ^(*n*)^, *y* ^(*n*)^, *ω*) are the complex data acquired by the transceiver placed at position (*x*, *y*) when emitting at the angular frequency *ω*; *D* is the distance from the aperture containing the transceivers to the centre of the volume where the computation is to be accomplished (see Fig. 3a); *c* is the speed of light in free space. The operator ${ {\mathcal F} }_{2D}$ denotes a bidimensional Fourier transform (FT) operating at each frequency on the spatial points (*x* ^(*n*)^, *y* ^(*n*)^, *ω*) and, consequently, it translates the points from the domain (*x* ^(*n*)^, *y* ^(*n*)^, *ω*) to the domain (*k*_*x*_, *k*_*y*_, *ω*), which can be finally translated into (*k*_*x*_, *k*_*y*_, *k*_*z*_) taking into account that ${k}_{z}=\sqrt{\mathrm{4(}\omega /c{)}^{2}-{k}_{x}^{2}-{k}_{y}^{2}}$. On the other hand, the operator ${ {\mathcal F} }_{3D}^{-1}$ denotes 3D inverse FT translating the input data from the spectral domain (*k*_*x*_, *k*_*y*_, *k*_*z*_) to the spatial domain (*x* ^(*n*)^, *y* ^(*n*)^, *z* ^(*n*)^). The function *W* is a window function that aims to reduce the secondary lobe level of the image at the expense of some resolution loss.

In this configuration, the lateral (cross-range) and depth (range) resolutions are given by^27^:

2 $$\begin{array}{ccc}{\delta }_{x}=\frac{cD}{2{f}_{c}{L}_{x}} & {\delta }_{y}=\frac{cD}{2{f}_{c}{L}_{y}} & {\delta }_{z}=\frac{c}{2B}\end{array},$$

where *f*_*c*_ is the central frequency; *L*_*x*_ and *L*_*y*_ are the dimensions of the scanner aperture; and, *B* is the frequency bandwidth. Equation (2) reveals that the lack of resolution due to a reduction in the scanner aperture size can be, at least partially, compensated by reducing the imaging distance.

It is relevant to observe that, after performing the bidimensional FT, the points are not equally spaced in the spectral domain (*k*_*x*_, *k*_*y*_, *k*_*z*_) preventing the application of efficient fast FTs. This issue is solved by interpolating to a regular grid in a operation usually referred to as *Stoltz interpolation* ^27^.

For each scanner position, the computed reflectivity is only accurate for the observation points close to the scanner. The main reason is the fact that the incident field illumination decreases with the distance from the scanner, so the scattered field is expected to be weaker (and therefore, noisy). In addition, only observation points under a given angular margin are illuminated by all the aperture elements due to the shape of the radiation pattern of the elements. This accuracy limitation together with the fact that In addition, the total observation volume to be filled after a relatively large number of observations can be large, as it will be discussed later, can be large motivates to restrict the reflectivity calculation. Consequently, the reflectivity is calculated only at to the observation points located in front of the scanner. In particular, only the points inside a square truncated pyramid in front of the scanner are considered (see Fig. 3a). However, the presented efficient formulation in equation (1) requires computing the reflectivity on a regular grid along a cuboid (local reconstruction volume in Fig. 3a) by using fast FTs. After that, only the points in the aforementioned hull (truncated reconstruction volume in Fig. 3) are kept.

## Reflectivity merge

The presented method computes the reflectivity based on a local coordinates system defined by the vectors ${\hat{x}}_{n}$, ${\hat{y}}_{n}$ and ${\hat{z}}_{n}$, where the first two vectors are aligned with the axis of the transceiver grid placed at the aperture of the scanner and whose origin *O* is at the centre of the transceiver grid as defined in (see Fig. 3a). The first step before merging the reflectivities is to translate them into a common system of coordinates. If the position and pose of the scanner with respect to a global system of coordinates are known, then the reflectivity for the *n*-th scanner position *ρ*_*n*_(*x* ^(*n*)^, *y* ^(*n*)^, *z* ^(*n*)^) can be translated into the global coordinate system (*x*, *y*, *z*) by:

3 $$[xyz]=[{x}^{(n)}{y}^{(n)}{z}^{(n)}]{\bar{\bar{R}}}_{n}+{\bar{T}}_{n},$$

where ${\bar{\bar{R}}}_{n}$ and ${\bar{T}}_{n}$ are the corresponding rotation matrix and translation vector.

In the presented problem, the use of a multiview setup with arbitrary positions of the scanner will result on overlap areas, which are covered by two or more scanner positions (see Fig. 3b). Therefore, a strategy for the combination of the reflectivities is required.

The two main approaches to merge different data of EM fields are *coherent* and *non-coherent* combination. Coherent combination is based on directly adding the complex values of the reflectivity so that the final image benefits from constructive and destructive interference. However, this method requires an accurate knowledge of all the involved parameters. When, as it will be shown later for the considered approach, positioning error is significantly larger than the working wavelength this family of methods is discarded.

If the number of uncertainties in the system is large, resulting in errors which cannot be neglected because they are in the wavelength range, non-coherent approaches are usually preferred. These approaches, though suboptimal, are more robust against inaccuracies in the knowledge of positions^28^. Among non-coherent techniques, the sum of only the magnitude of each pixel is the most widespread approach (e.g., ref. 29). However, this method is not expected to provide good results for the considered multiview problem as it would result in an artificially strong reflectivity in those areas that are covered by two or more scanner positions. In order to avoid this problem, the use of the maximum reflectivity observed for each pixel is proposed:

4 $$\rho (x,y,z)=\,{\rm{\max }}\{|{\rho }_{1}(x,y,z)|,|{\rho }_{2}(x,y,z)|,\ldots ,|{\rho }_{N}(x,y,z)|\},$$

where *N* is the number of views. This approach has the advantage that only the strongest values, which are usually related to observations with high signal to noise ratio, are retained without highlighting overlap areas. The main disadvantage is that it does not take full advantage of the different observations to produce a clearer image.

## Computer vision techniques

In order to estimate the scanner pose and positions as well as to enable an estimation of the optical 3D model, pictures are taken from, at least, the same positions from where the EM data has been acquired. In practice, it is recommended to include also some intermediate pictures to increase the degree of overlap.

Pictures are processed by resorting to standard SfM^30^, which comprises the following steps. First, the relevant points of each image are identified and descriptors are computed by using the scale-invariant feature transform (SIFT). Second, the descriptors between sequential images are compared to find the matching points. Third, the global three-dimensional 3D position of the matching points is found by triangulation so that a *sparse reconstruction* of the 3D optical model is found. At this step, camera position and poses are also retrieved.

Two additional steps are possible depending on the desired quality of the 3D optical model. Once the camera motion is estimated, it is possible to establish more accurate matches between additional points by using the epipolar geometry constraints^31^ yielding a *dense reconstruction*. Finally, it is possible to create a solid triangle mesh from the dense point cloud by using Poisson surface reconstruction^32^.

## Results

In order to validate the approach, two examples are presented next. The first example is devoted to illustrate the performance of merging multiple views by means of equation (4). For this purpose, a computational simulation is performed in order to generate multiview data. The second example pursues to illustrate the capabilities of a system in a real environment. In this latter case, the scanner aperture, which would be able to perform real-time fully electronic imaging in a final system, is emulated by means of raster scanning with a single general-purpose sensor.

**Figure 4:**
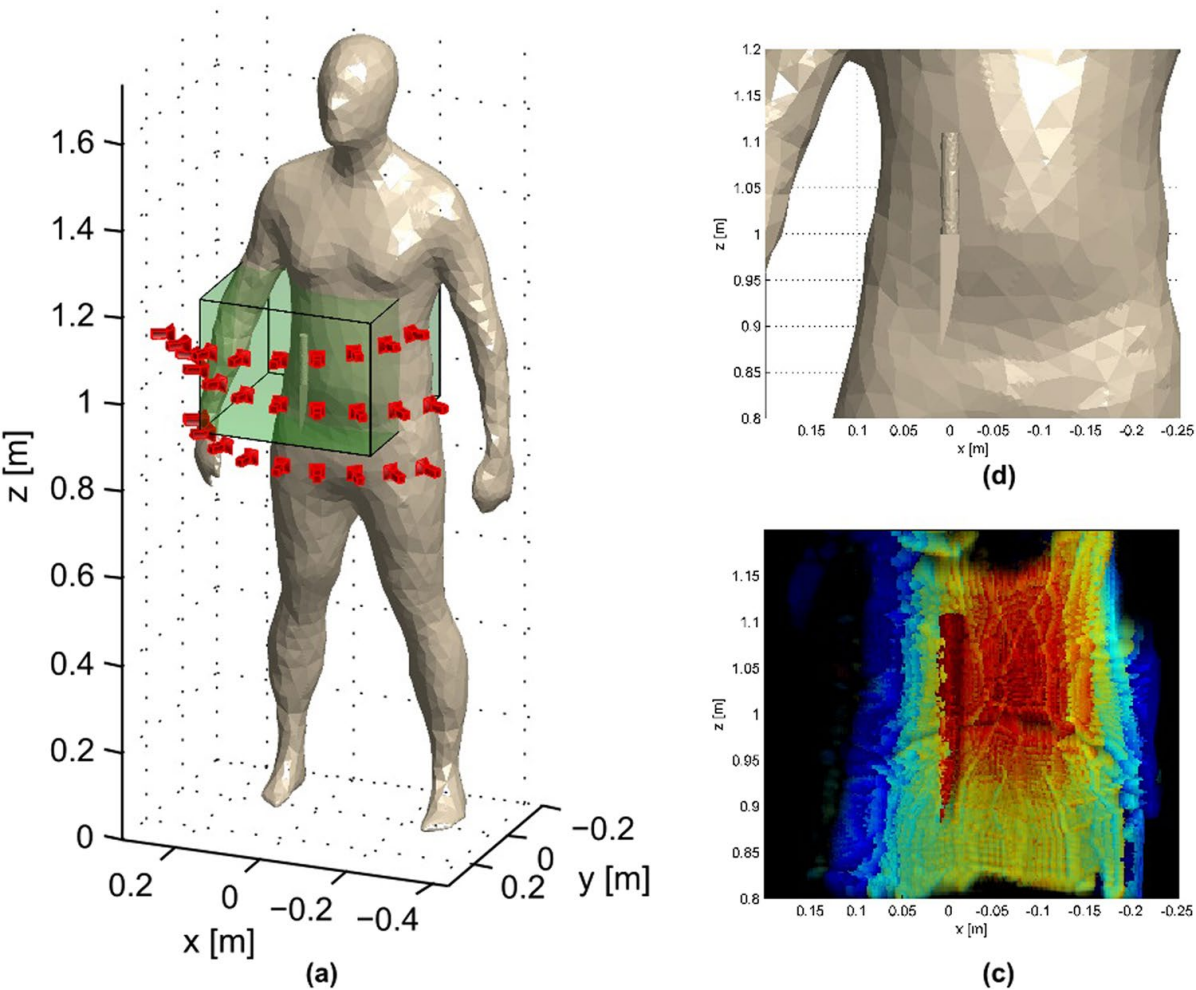
Simulation example comprising 27 scanner positions: (**a**) 3D model, camera positions and reconstruction volume; (**b**) detail of the knife; (**c**) mm-wave image considering all the positions.

Thus, let us first consider the triangle mesh model depicted in Fig. 4a where a knife-like geometry is placed close to the waist line. The simulation is performed considering the scanner acquires data at the 27 positions shown in Fig. 4a.

The scanner consists of a square aperture with 80 × 80 transceivers equally spaced by half-wavelength at the maximum frequency, yielding a 15 cm × 15 cm aperture size. In this example, 24 frequencies ranging from 75 GHz to 79 GHz are considered. This frequency band has been is widely used for automotive radars resulting in very cost-effective components.

In order to build an image from the 3D reflectivity *ρ*(*x*, *y*, *z*), a procedure similar to the one described in ref. 11 is considered. Thus, a surface is firstly constructed by considering the maximum along the *y*-axis:

5 $$S(x,z)={\rm{\arg }}\,{{\rm{\max }}}_{y}|\rho (x,y,z)|$$

and, after that, an image is constructed by using a colour-scale associated to depth. In addition, the brightness of each pixel is weighted by its reflectivity at the considered surface point.

**Figure 5:**
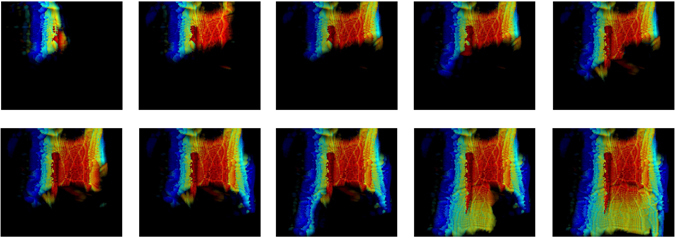
Accumulative Cumulative image for the simulation detailed in Fig. 4 after 3, 6, 8, 10, 13, 16, 18, 20, 23 and 26 scanner positions, respectively.

Cumulative results for different intermediate positions are shown in Fig. 5 while the final image, after merging 27 positions, is shown in Fig. 4c together with a detail of the view around the reconstruction area (Fig. 4b). Despite the relatively small scanned area, the knife can be easily detected.

It is relevant to note that two artefacts appear in the image. First, there is a small gap along the torso at approximately *z* = 1 m. This effect is due to the scanning path, since it does not completely cover this area. However, this effect can be removed by adding some extra positions. The second visible artefact is the presence of some yellow pixels in the image. This yellow pixels, in contrast to the red colour which corresponds to the depth of the knife, are due to the 3D representation into a two-dimensional projection given by equation (5). Since the simulation considers only perfect electric conductors to alleviate the simulation cost, the reflectivity of all the materials is expected to be the same. Consequently, it is possible that, after the numerical processing, some pixels behind the knife (and corresponding to the torso) have a slightly higher reflectivity computed values and, therefore, they are represented instead of the pixels related to the knife.

**Figure 6:**
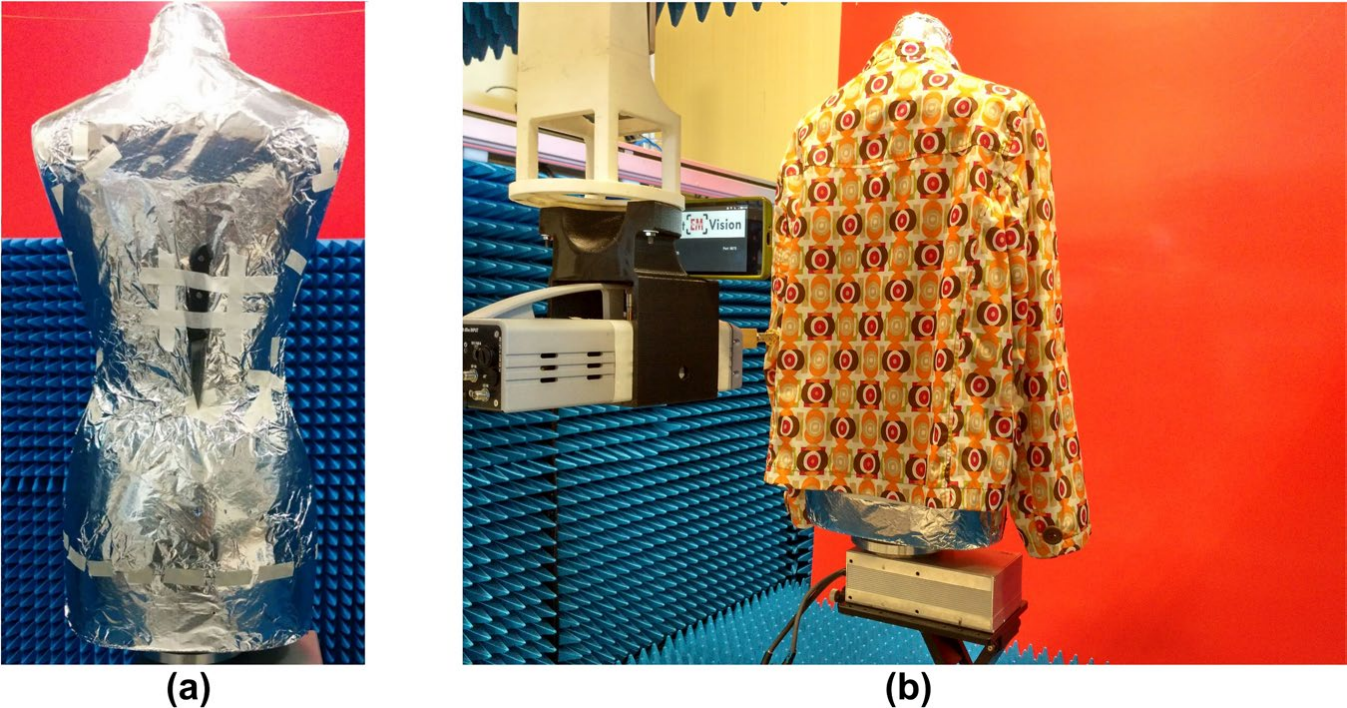
Measurement setup comprising a mannequin with an attached knife on a rotary platform, an optical camera and the mm-wave sensor used to implement the scanner aperture installed on the three-axes linear stage: (**a**) without coat; (**b**) with coat.

Next, the approach is validated by measurements. For this purpose, a mannequin torso with an attached knife has been considered (see Fig. 6a). The torso of the mannequin is covered by aluminium foil since it provides a fair approximation of the human skin at mm-waves. It is important to note that this entails a more difficult case than considering a more realistic material to model the skin since the contrast between some parts of the knife (mainly the blade) and the background torso is reduced. The mannequin is dressed with the coat shown in Fig. 6b.

A setup equivalent to moving the scanner along 14 different positions is considered (see supplemental material for further details). For each scanner position, a general purpose module is moved along a grid of 115 × 115 positions separated 1.4 mm. Thus, a scanner aperture of size 15.96 × 15.96 cm^2^ is emulated.

In order to estimate the scanner positions, a smartphone with a customized image acquisition software is attached to the setup as shown in Fig. 6b. After processing the pictures with the SfM software^33^, the position estimation accuracy is of approximately 1 cm.

Although the camera perceives that it is rotated around the mannequin, due to the particularities of this setup the background is seen as static. This artificial situation can mislead the 3D optical model estimation and, therefore, it will have an impact in the positioning accuracy. In order to mitigate undesirable effects, a flat monocolour panel has been placed behind the mannequin so that the multiview optical algorithms are not able to find key points in the background. Possible alternatives to avoid the use of this panel will be discussed later.

The scattered field is measured in the entire W-band (75 GHz–110 GHz) with 401 frequency points for validation purposes, but only 115 frequency points from 95 GHz to 105 GHz are retained to produce the final images. Assuming a distance to the target of 30 cm, this setup provides a lateral resolution of 2.8 mm and a depth resolution of 1.5 cm^27^.

The iterative computation of the 3D mm-wave model is shown next. In this case, only the aforementioned frequency band from 95 GHz to 105 GHz is considered.

**Figure 7:**
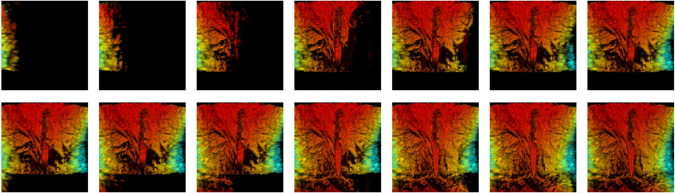
Cumulative image for the setup described in Fig. 6.

**Figure 8:**
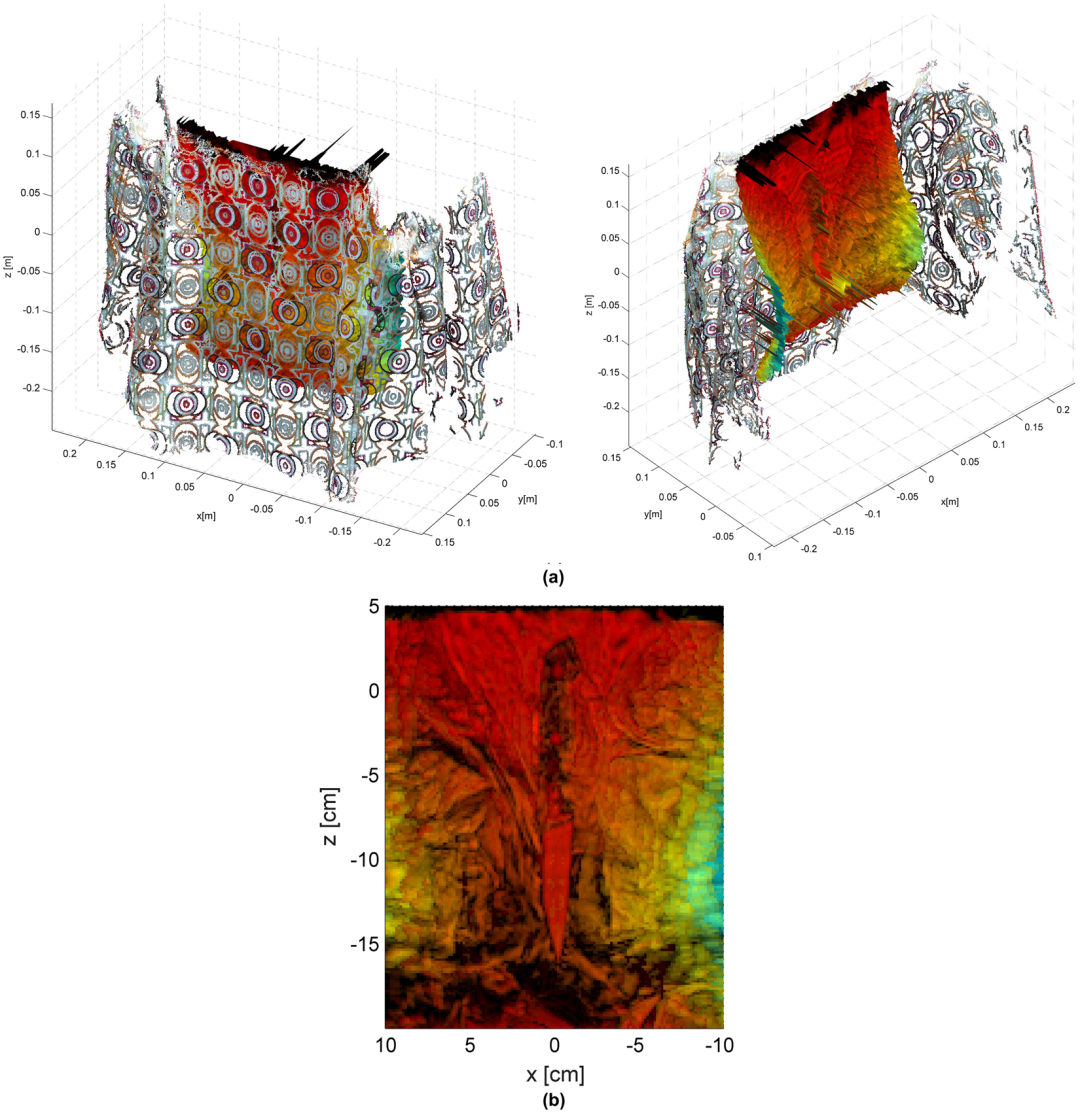
Mm-wave image considering all the scanner positions: (**a**) 3D model superposed with the dense point cloud extracted from image from two different perspectives; (**b**) projected mm-wave image onto XZ plane.

Sequential steps for the cumulative reflectivity are shown in Fig. 7 where the partial reflectivities are merged to yield a global mm-wave model where the knife can be perfectly seen. Since the 3D model from optical images and the mm-wave model are computed using the same coordinate system, it they can be superposed as shown in Fig. 8. The video attached in the supplemental material provides a further inspection as well as the solid triangle mesh model computed by Meshlab^34^. The mm-wave images are accurately merged and they match well the optical model, providing a very valuable information to detect the hidden knife.

**Figure 9:**
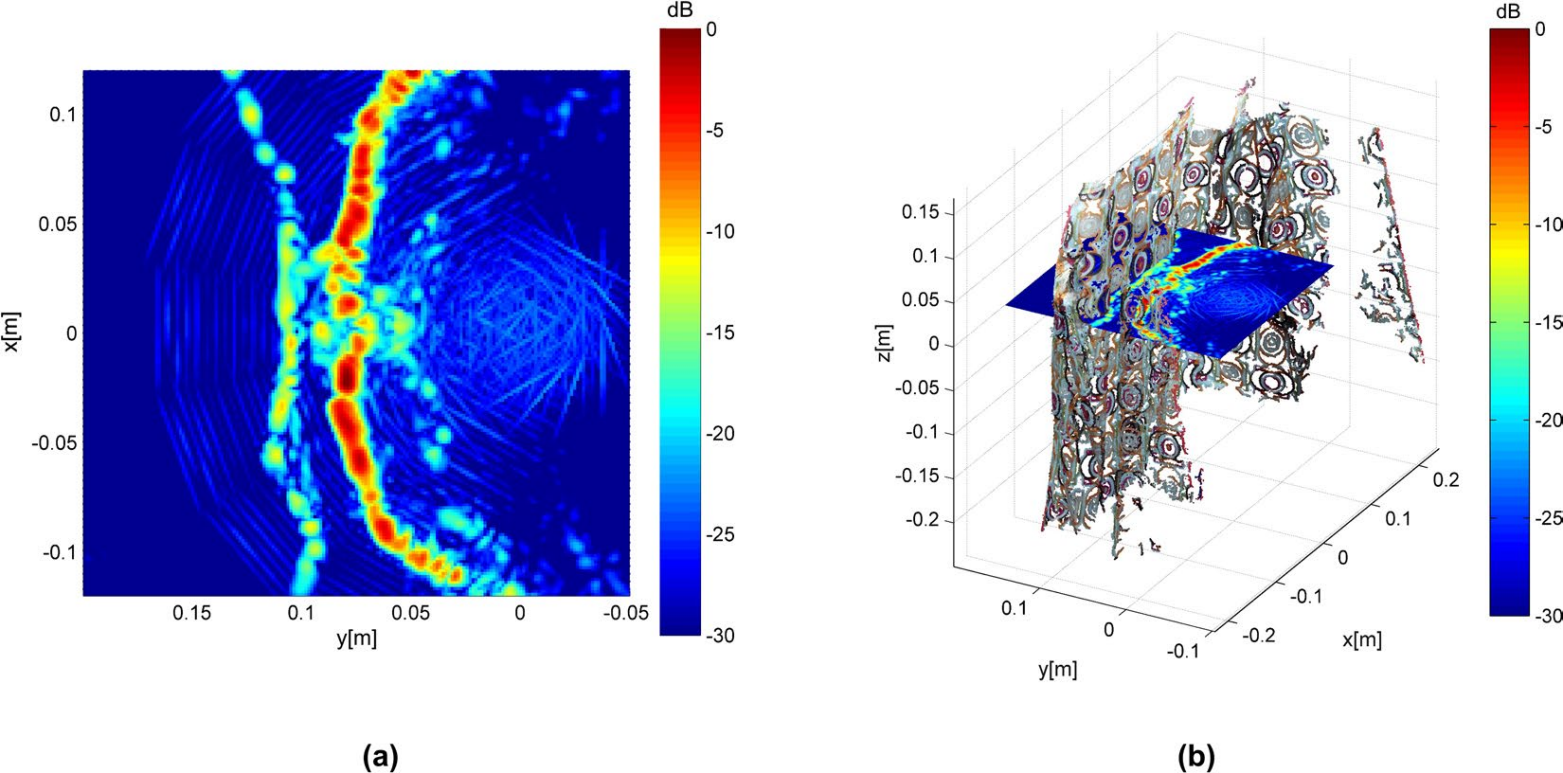
Reflectivity computed for a cut at *z* = 0 cm: (**a**) overview; (**b**) superposed dense point cloud obtained from optical images.

In order to further validate the proper behaviour of the proposed techniques, let us consider Fig. 9a which corresponds to a cut of the reflectivity in the *x*-*y* plane. These results are obtained with the entire W-band measurements to achieve the best possible depth resolution (4.3 mm). A clear contour, corresponding to the mannequin torso, is detected. In addition, a second contour with a reflectivity that is between 10 dB and 15 dB below the reflectivity of the strongest contour is also found at *y* = 0.1cm approximately. The superposition of the 3D model from optical images as shown in Fig. 9b clearly reveals that this second contour matches perfectly the coat shape. The dense point cloud model computed by VisualSFM^33^ has been used in this image.

## Discussion

Multiview mm-wave imaging with small aperture devices has been demonstrated in this paper. This technology is expected to enable real-time imaging benefiting of the penetration capabilities of mm-waves. For example, it can be useful in the security area, where current devices are bulky and can be perceived as frightening. Furthermore, the presented approach can scan locally in contrast to current scanners where the image is never generated due to privacy concerns introducing an abstraction layer so that the system is image-free. For example, it can be useful in the security area, in which booth devices can provide a quick and accurate image with enough spatial resolution to detect objects of interest but they have difficulties, subject to an intense research currently, to generate an image containing information from a large number of angles. Furthermore, booth systems are usually bulky and can be perceived as frightening. Thus, the proposed local scanning technique by merging multi-view information is a good candidate to provide complementary information in this area.

Although the system has been tested for static targets, the results are expected to be robust in case of targets subject to small movements, like respiration. On one hand, according to the state of the art, a fully electronic acquisition can be performed in milliseconds and, therefore, the target can be considered as static during each acquisition. On the other hand, the proposed multiview merge is based on incoherent combinations and, therefore, it does not require coherence between different acquisitions. Thus, the performance is are expected to be similar to the results achieved in the past for 3D modelling based on SfM. Thus Hence, although the 3D model could suffer from global artefacts, the model for local areas is expected to be accurate enough so that very valuable information can still be inferred. Similar conclusions can be drawn for the case of non-collaborative targets as each local image is expected to be accurate although the global model, after stitching the multiple views, can be deformed but still able to provide useful details.

There are several points that will be considered to be improved. First, the proposed architecture requires a dense aperture populated by equally spaced monostatic transceivers. To overcome this drawback, sparse designs based on a multistatic aperture are currently under consideration so that the number of transmitters and receivers, as well as their density, is reduced. Furthermore, this is expected to release enough space in the aperture so that the camera can be included inside the aperture avoiding the need of introducing offsets between the coordinates systems of coordinates of the optical and mm-wave models.

In the current proof-of-concept implementation, an optical camera has been considered with a two-fold purpose: i) estimate the arbitrary positions where the scanner has performed the mm-wave data acquisitions; ii) provide an optical model. Although it enables a budget solution that meets well with current multiview technology, this is expected to be improved in the future by including RGB-D cameras. This kind of cameras provide not only the colour of each pixel but also the its depth yielding a coloured point cloud that can be used for positioning^35^. This is expected to improve the resolution in cases with flat textures where the number of keypoints from optical images is drastically reduced yielding a positioning system that is independent of the subject attire. Alternatively, RGB-D cameras can be used as a conventional camera but with the ability to filter the pixels beyond a given distance avoiding the need of using neutral backgrounds. Furthermore, these depth cameras can be based on either structure-from-light or time-of-flight technology, which are both able to work with independence of the illumination as they use their own infrared sensors and, consequently, this approach is expected to be robust against illumination changes and light shadows.

The use of other accurate positioning systems such as laser trackers can also be of great interest as they provide an accuracy smaller than the wavelength. Consequently, they would open the possibility of coherent combination yielding improved resolution for the mm-wave model. However, that technology would significantly increase the cost of the overall system and it would require the deployment of fixed anchors, reducing the portability of the system.

## Methods

### Optical images acquisition and processing

The optical images are processed by means of the software VisualSFM^33^, whose kernel is open source. Standard configuration is used to detect the point descriptors by SIFT as well as to generate the sparse points. Dense point cloud is also computed by VisualSFM with additional module CMVS^36^. The solid mesh shown in the supplementary information is generated by using the open-source software Meshlab^34^ using as input the dense points computed by the CMVS.

In order to take the pictures automatically, a smartphone equipped with a camera is employed. The relevant features of the camera are a 8 megapixels sensor with 1.4 m of pixel size, an aperture of f/2.2, an equivalent focal length of 28 mm and autofocus capability. The smartphone is running an *ad hoc* dedicated software under the operative system to automatically capture the images.

### Mm-wave acquisition system

Field acquisitions are made by means of an Agilent N5247A PNA-X with a ZVA-Z110 frequency extender module from RPG with a multiplying factor of x6 for the local oscillator and RF inputs. The input power for both inputs was set to +7dBm and the intermediate frequency was set at 270 MHz. The acquisitions are made for 401 frequency points between 75 GHz and 110 GHz and a time gating from 1.15 ns to 3 ns is applied to remove the antenna inner reflections. The measurements are made in a semi-anecoic measurement range provided with a multi-axis linear positioner and a rotary positioner from IAI Robots with positioning repeatabilities of 10 m and 5millidegrees, respectively.

### Synthetic aperture imaging processing

After finishing all the scattered field acquisitions and scanner position estimation, the reflectivity was calculated offline. Before performing the computation, the term ${e}^{-jD\sqrt{\mathrm{4(}\omega /c{)}^{2}-{k}_{x}^{2}-{k}_{y}^{2}}}$, which does not depend on the scanner position, was precomputed. The window function *W*(*x*, *y*, *ω*) was set as a three-dimensional Hamming window.

Reflectivity computation algorithm was implemented using Matlab. The first example, corresponding to computational simulations, was run on a workstation equipped with one Intel Core i7 3820, 64GB of RAM and an Nvidia GTX 770 with 2GB of VRAM. For the second example, corresponding to measurements on a mannequin, the problem could not be solved on the GPU due to the larger size of the problem in terms of frequency and transceivers. Thus, a computational server equipped with 2 Intel Xeon E5-2650v3 and 256GB of RAM was used.

In the first example, the observation volume was a cuboid ranging from [−25, 20] × [−20, 20]×[80, 120]cm according to the coordinate system shown in Fig. 4a. The reflectivity was computed on a regular grid of 211 × 211 × 238 observation points inside the described cuboid. The bottom base of the pyramidal frustum was placed at 14.1 cm from the scanner aperture and it had a dimension 22.6 cm, whereas the large base, placed at 23.5 cm from the small base, had a size of 75.2 cm. It resulted in a number of observation points between 2130816 and 2790576 depending on the scanner position. The average time to compute the reflectivity for each position of the scanner was 211 ms on the aforementioned computational server equipped with a GPU.

In the second example, the reflectivity was computed in a cuboid ranging from [−12, 12] × [0, 20] × [−15, 15] cm. Due to the finite beamwidth of the considered antennas, the frustum was slightly reduced with respect to the simulation example and it comprised two bases of size 23.1 cm and 28.5 cm where the small one is separated 16 cm from the scanner aperture and the large one is separated 10.3 cm from the small one. Reflectivity computation time for this second problem results in an average time of 1.18 s when the secondly described computational server is used.

### Computational simulation

EM simulations for the presented examples first example are were accomplished using the software Feko 7.0^37^. Among the available simulation methods, “Large Element Physical Optics” has been employed since full-wave analysis is not feasible due to the high time and computation demands. The transmitting and receiving elements were modelled with a cos^*q*^ radiation pattern with *q* = 1.

## Electronic supplementary material

Supplementary Information

Iterative reconstruction
