# Supplementary material for: Multiview three-dimensional reconstruction by millimetre-wave portable camera
Source: Sci Rep. 2017 Jul 25;7:6479. doi: 10.1038/s41598-017-06475-7 (PMC5527015; doi:10.1038/s41598-017-06475-7)
Supplement: Supplementary file 1 — Supplementary Information [file 41598_2017_6475_MOESM1_ESM.pdf]

Multiview three-dimensional reconstruction by millimetre-wave portable camera

Jaime Laviada[1,*], **Ana Arboleya**[1], **Yuri Álvarez**[1], **Borja González-Valdés**[2], **and Fernando Las-Heras**[1]

[1]Universidad de Oviedo, Dept. Ingeniería Eléctrica, Gijón, 33203, Spain
[2]Universidad de Vigo, Dept. Teoría de la Señal y Comunicaciones, Vigo, 36310, Spain
[*]jlaviada@tsc.uniovi.es

ABSTRACT

This document provides the details of the implementation carried out to emulate the movement of a square aperture scanner around a small area of a human torso. The measured data are also provided together with a description of the file formats.

Setup description

In order to mimic the movement of a scanner aperture around a human body with a single Tx/Rx module, a combination of three linear axes and rotary movements has been used. For this purpose, the mannequin torso, covered by aluminium foil, is placed on a rotary platform whereas the mm-wave Tx/Rx module is mounted on a three-axes positioner. The scanner aperture is achieved by moving the positioner along the equally spaced points of the aperture, by means of the three-axes positioner, while the movement along the body is simulated by rotating the mannequin torso.

A complete description of the measurement system and radiofrequency (RF) instrumentation can be found in the appendices of[1]. It is worth mentioning that the positioning accuracy of the linear stages is $\pm 10\,\mu$m while for the rotary positioner is $\pm 0.005°$, thus the effect of positioning errors is disregarded and accuracy of results is expected to be similar to the one obtained with a fully electronic aperture array as the one intended to use as a final prototype.

For the validation example shown in the paper, 14 scanner positions distributed in two different horizontal cuts separated by a vertical distance of 10 cm are considered. Each cut of scanner positions comprises 7 different scanner acquisitions at a distance of 20°. In an equivalent system, in which the scanner would be moved along the target under test, these positions would be distributed as shown in Figure 1. Red dots represent the equivalent acquisition points of the scanner aperture, grouped for each of the 14 positions while green dots represent the centre of the scanner where the optical camera would be placed.

In order to define the involved parameters, let us consider the central rotation position for one of the two considered cuts as shown in Fig. 2a. For this position, the x- and z-axes are defined parallel to the edges of the square aperture. The centre of the

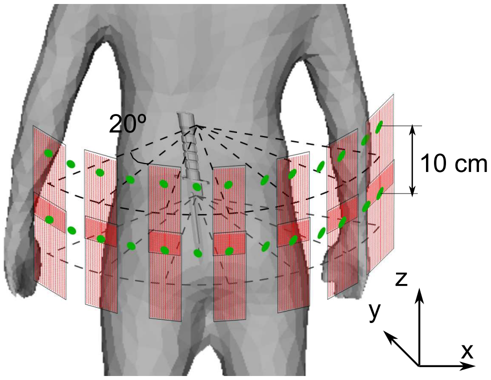

Figure 1. Schematic view of the acquisition setup with 14 scanner positions. The red dots represent the acquisition points for each position of the scanner while green dots illustrate the position of the optical camera. The acquisitions are distributed into two horizontal cuts (i.e., contained in the xy plane) and there is a virtual overlap between the acquisitions at the two different z levels.

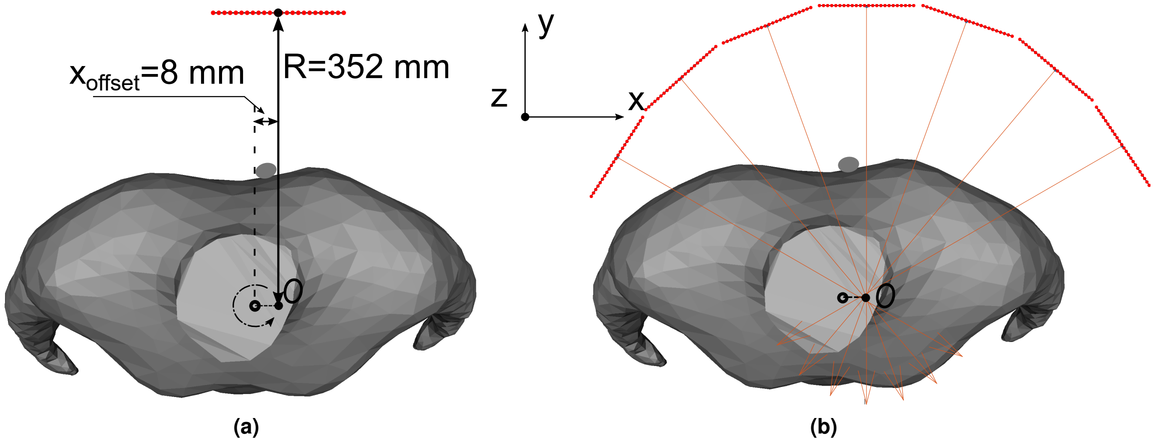

Figure 2. Overhead view of the setup comprising a mannequin with an attached knife on a rotatory platform : a) setup measurement ; b) equivalent setup measurement.

scanner for that central position has an offset of 8 mm and 352 mm with respect to the centre of rotation for the *x*- and *y*-axes, respectively (see Fig. 2a).

Taking into account the previous definition of the system of coordinates, the rest of positions can be defined assuming that the body is fixed and the aperture is moved as shown in Fig. 2b. The corresponding theoretical positions for this setup are gathered in Table 1.

A smartphone with a home-made software is attached to the setup (see Fig. 6b in the main paper body) to estimate the scanner positions. Due to physical restrictions, the camera is placed with an horizontal and a vertical offset with respect to the centre of the scanner aperture. Although the vertical offset could be compensated when taking the pictures by properly moving the positioner, the horizontal offset is only compensated partially due to physical restrictions yielding a final offset of 8.1cm. This offset is compensated later during the estimation of the scanner positions.

In order to increase the degree of overlap among pictures, rotation steps of 5° are considered yielding a total of 50 pictures. Besides, 3 extra pictures separated by 1 cm are also taken in order to estimate the scale factor of the model. In practice, this scale factor can be estimated by considering at least two cameras separated by a known distance.

Although the camera perceives that it is rotated around the mannequin, due to the particularities of this setup the background is seen as static. This artificial situation can puzzle the 3D optical model estimation and, therefore, it will have an impact in the positioning accuracy. In order to mitigate undesirable effects, a flat monocolour panel is placed behind the mannequin so that the multiview optical algorithms are not able to find key points in the background.

In this setup, a systematic error is expected for the rotation matrices estimation due to misalignment between the camera and the scanner system of coordinates. This error can be compensated by a proper calibration. In the presented implementation, the rotation matrix are decomposed into yaw, pitch and roll so that the average value of the last two angles, which should be ideally zero for the considered movements, are subtracted.

The estimated positions are shown in Table 1. Only the 14 positions from pictures taken at the same positions as the scanner positions are provided. As it is seen, the system provides an accuracy in the order of 1 cm.

Data format

For the sake of reproducibility, the measurement data can be downloaded from `http://www.tsc.uniovi.es/~jlaviada/CV_and_mmW/MeasData.zip`. In the compressed folder with the data, a different file for each of the 14 scanner positions is provided under the file names `1.txt`, `2.txt`, etc. following the same order as the positions described in Table 1.

Data is presented as follows, the three firsts rows corresponds to the header of each file detailing the parameters that are acquired as well as their format. In this case, the S_{11} parameter is saved in phase (degrees) and amplitude (dB) for the whole set of frequencies. Then, each row of the data files contains the information of the scattered field for each of the considered spatial points of the scanner aperture as defined in the fourth row of the header. First column is a dummy column set to one, columns one to four contain the coordinates for the spatial acquisition point of the raster scan, while column five shows the angle of the rotary platform in degrees. After the grid information, the phase data for all the frequencies, followed by the amplitude information, can be found.

Camera position						Camera attitude					
x [cm]	$\hat{x}$ [cm]	y [cm]	$\hat{y}$ [cm]	z [cm]	$\hat{z}$ [cm]	γ [deg.]	$\hat{\gamma}$ [deg.]	α [deg.]	$\hat{\alpha}$ [deg.]	β [deg.]	$\hat{\beta}$ [deg.]
30.91	30.21	18.31	17.61	0	-0.36	60	60.44	90	88.77	0	0.46
22.83	22.19	27.50	26.56	0	-0.12	40	40.15	90	89.53	0	0.68
12.10	11.78	33.37	32.22	0	0.04	20	20.04	90	89.88	0	-0.05
0.00	0.00	35.23	33.67	0	0.10	0	0	90	90	0	0
-12.00	-11.4	32.83	31.20	0	0.15	-20	-20.12	90	90.07	0	0.08
-22.46	-21.33	26.47	24.96	0	0.21	-40	-40.25	90	90.21	0	0.21
-30.11	-28.41	16.92	15.68	0	0.22	-60	-60.27	90	90.23	0	0.32
30.91	29.76	18.31	17.40	-10	10.16	60	60.16	90	90.44	0	-1.16
22.83	22.75	27.50	26.98	-10	10.30	40	40.40	90	90.47	0	-0.42
12.10	11.83	33.37	32.23	-10	10.30	20	20.02	90	90.42	0	-0.056
0.00	0.11	35.23	33.58	-10	10.29	0	0.02	90	90.41	0	0.11
-12.00	-11.47	32.83	31.29	-10	10.30	-20	-20.12	90	90.40	0	0.28
-22.46	-21.35	26.47	24.93	-10	10.29	-40	-40.32	90	90.39	0	0.44
-30.11	-28.44	16.92	15.65	-10	10.23	-60	-60.30	90	90.29	0	0.57

Table 1. Theoretical camera positions (x, y, z) and attitude angles described by yaw (γ), pitch (α) and roll (β). Estimations from optical images are denoted by $\hat{\cdot}$.

Parameter	Format	Sweetype								
S11	PHAS	LIN								
S11	MLOG	LIN								
0	x[mm]	y[mm]	z[mm]	phi[deg]	PHAS-S11-f1	...	PHAS-S11-f401	MLOG-S11-f1	...	MLOG-S11-f401
1	1416.681	709.737	100.572	270	-10.536	...	-144.956	-25.816	...	-27.924
1	1416.681	711.137	100.572	270	-10.123	...	-144.485	-25.808	...	-27.932

Table 2. Example of the first rows of a data file for each of the considered scanner positions.

The coordinates of the measured data in the provided files are expressed in the the positioning system coordinates described in[1]. In this system the z-axis, is inverted with respect to regular conventions since it is defined as $\hat{z} = -\hat{x} \times \hat{y}$. In the measurement, the scan aperture was set to be in the yz plane. In order to minimize the raster scan time, the grid was measured in a zigzag path starting by the point with minimum y and z coordinates and moving towards $+\hat{y}$. The angular variation ranges from 210° to 330° in steps of 20°, being the central position at 270°. The rotation axis was at $x = 1064.4$ and $y = 781.5$. For each angular position the scanned aperture consist of 115×143 points sampled with 1.4 mm step but the first and last 14 points along the z-axis are discarded to yield the final aperture with 115×115 points.

The first rows of a sample file are shown in Table 2 including the header section and a couple of rows with data in order to further describe the file format.

References

1. Arboleya, A. *Novel XYZ scanner-based radiation and scattering measurement techniques for antenna diagnostics and imaging applications*. Ph.D. thesis, Universidad de Oviedo (2016). Online available: http://hdl.handle.net/10651/40222.
